# A Business Model Framework for Software as a Medical Device Startups in the European Union: Mixed Methods Study

**DOI:** 10.2196/67328

**Published:** 2025-05-23

**Authors:** Rahel Sophie Martjan, Sascha Noel Weimar, Orestis Terzidis

**Affiliations:** 1 Institute for Entrepreneurship, Technology Management and Innovation (EnTechnon) Karlsruhe Institute of Technology (KIT) Karlsruhe Germany

**Keywords:** digital health, European Union, Medical Device Regulation, MDR, software as a medical device, entrepreneurship, startup, business model, business model framework, components

## Abstract

**Background:**

With the introduction of Regulation (EU) 2017/745, also known as the Medical Device Regulation (MDR), startups aiming to develop software as a medical device (SaMD) in the European Union are confronted with stringent and complex regulations, and many of them struggle with them. Complying with the MDR is a costly, time-consuming endeavor requiring expertise and substantial financial resources. However, it opens the door for new revenue models, such as reimbursement pathways. Consequently, the MDR significantly shapes the business model of startups. Early on, the regulation needs to be considered for business modeling to survive the conformity assessment process financially. Business model frameworks are tools that reduce complexity by focusing on the key aspects of a business model. Thereby, the risk of overlooking essential elements can be minimized. A framework directly integrating the MDR could alleviate the intricate circumstances in which SaMD startups are entangled.

**Objective:**

This study focused on deriving a business model framework for startups aiming to develop SaMD under the MDR. With the framework, we strived to facilitate business modeling for SaMD startups.

**Methods:**

The study is based on a 3-step approach. First, a systematic literature review was carried out, resulting in a concept matrix and an overview of business model frameworks developed and applied in the digital health sector. Subsequently, 13 interviews were conducted with experts and startups in the SaMD industry. On the basis of the literature analysis and expert interviews, along with the MDR requirements for SaMD, we developed the SaMD business model framework.

**Results:**

The SaMD business model framework consists of 13 interrelated components, with the MDR being a pivotal component. The centerpiece of the framework is the “regulatory value arc,” which is the trio of intended purpose, value propositions, and customer segments. For each component, valuable input is provided, making the framework tailored to startups aiming to develop SaMD.

**Conclusions:**

The findings highlight the crucial role of regulations in business modeling. Notably, the MDR is a central regulation for startups aiming to develop SaMD. The study uncovers the impact of the MDR on business modeling for SaMD startups. Our research provides a framework that integrates this regulation, thereby reducing its complexity and facilitating startups in deriving a sustainable business model. Concurrently, this research integrates the domains of business modeling and the MDR. Therefore, it contributes to the academic discourse in both fields and addresses a notable gap in the existing literature.

## Introduction

### Motivation and Objectives

Regulation (EU) 2017/745, also known as the Medical Device Regulation (MDR), is highly debated. The focus of the literature concerning the MDR mainly centers on its challenges and complexity [1,2]. The MDR is associated with strict regulatory requirements [3], the involvement of multiple stakeholders [4], delayed market access [5], ambiguity [6], and a financial burden [7]. The expenses related to the MDR include costs for personnel undertaking activities to meet the requirements of the MDR. In addition, the setup, maintenance, and certification of the quality management system (QMS) are significant cost drivers. For products exceeding risk class I, fees for the conformity assessment conducted by a notified body must be considered [8]. A significant cost driver is the clinical investigation [9]. It requires a substantial amount of time, expertise, and potential fees for the study site. The MDR considerably impacts the business model of software as a medical device (SaMD) startups, which are challenged to incorporate the regulation into their business model. SaMD describes software intended to be used for at least 1 medical purpose, performing these purposes without being part of a hardware medical device [10]. SaMD ranges from solutions primarily used by patients for treating medical conditions, such as depression, to solutions that support physicians in diagnosing and preparing treatment plans [11]. SaMD also includes platforms used by physicians to monitor patients’ medical conditions [11]. Simpler apps used by patients are often associated with lower risk compared to complex diagnostic tools used by physicians. For lower-risk devices, the regulatory burden under the MDR is reduced, such as in the postmarket surveillance process [12] or the involvement of a notified body [12,13].

The requirements of the MDR apply to all medical device manufacturers irrespective of their company size [14]. Consequently, they seem to disproportionately impact small-to-medium enterprises and startups [9]. Often inexperienced and possessing limited resources, they face considerable barriers [9]. Startups struggle with the complexity of the requirements and their lack of regulatory understanding [12,15]. In addition, startups at an early stage find it difficult to grasp the implementation of a QMS that requires structured processes and extra effort [14]. Finally, the clinical investigation, if required, is especially challenging for many startups due to its financial costs and time investments [14,15].

Ironically, startups foster more radical innovation [16] and are, therefore, a driving force in the SaMD industry. The flexibility and adaptability of startups are a door opener for digital innovations [17]. The strict and complex MDR, on the one hand, and the innovative and flexible culture of startups, on the other, create the impression of a clash between 2 distinct cultures [14]. Understandably, this confrontation puts startups with obstacles many cannot tackle, thereby jeopardizing innovations in the SaMD industry. According to Stern [18], industry regulatory requirements are frequently linked to firms postponing or diminishing market entry due to increased time and costs. For smaller firms, it is more challenging to act as pioneers in medical device markets due to the relatively higher costs and the greater financial constraints compared to established companies [18]. Nevertheless, the MDR plays a crucial role in shaping industry standards for medical devices in the EU and globally. Bradford [19] describes the power of the EU to project its regulatory standards to the rest of the world as the “Brussels effect.” For example, Canada based its medical device regulations on the standards of the EU Medical Device Directives [20], the predecessor of the relatively new MDR. The MDR aligns with international industry standards, facilitating market access beyond the EU.

Although the literature on the challenges of the MDR, specifically for SaMD startups, is profound [1-3], possible solutions to mitigate them are missing. Business model frameworks are tools that facilitate business modeling. By focusing on the key aspects only, they reduce complexity while ensuring that no crucial business components are missing. Consequently, especially in complex settings, business model frameworks are perceived as powerful tools. The situation in which startups aiming to develop SaMD are embedded is intricate, with the MDR being a significant challenge. A business model framework that directly incorporates the MDR might assist them in deriving a sustainable business model and mitigating the inherent complexity of the MDR.

In the literature, a couple of business model frameworks can be found. A widely recognized [21] and used tool [22] is the Business Model Canvas by Osterwalder and Pigneur [23]. Having no industry focus makes it a widely applicable but unspecific tool for business modeling. According to Albert and Van der Auwermeulen [24], general business model frameworks, such as the Business Model Canvas, do not sufficiently account for the specifics of the health care sector. The nuances of the health care sector require a specialized business model framework [24]. Therefore, there are increasing attempts to develop health-specific frameworks. One example is the framework for the telehealth sector by Velayati et al [25], which is based on interviews suggesting key components of a telehealth business model. Mueller [26] developed a mobile health business model framework that considers regulations. However, regulation is only included as a component and is not specified further. Outside of the digital health industry, regulations have been discussed. For example, Lindgren [27] researched the impact of General Data Protection Regulations on business models, but did not derive a business model framework to work with. Ademi et al [28] focused on the development of sustainable business models due to increased regulations and changing customer priorities. Still, literature regarding business modeling in regulated markets is in its infancy. More work is needed to understand the interplay between business models and regulations, particularly in highly regulated industries. To address this research gap, the primary objective of our work is to develop a comprehensive business model framework tailored for startups venturing into the development of SaMD in the EU. To achieve this pivotal goal, we formulated 3 research questions (RQs):

1. Which business model components and frameworks are mentioned in the digital health literature?
2. Which aspects of SaMD business model are considered important by SaMD startups and experts?
3. How can the results be synthesized into a conceptual framework considering the MDR?

The conceptual business model framework developed in this work provides a condensed solution for startups developing SaMD. The framework incorporates the key components these startups need to consider while directly integrating the specifics of the MDR. Especially startups in the early stage tend to overlook key aspects [14]. A business model framework can be instrumental in this context. Our work can assist SaMD startups in successfully entering the SaMD industry in the EU with a sustainable business model. The hurdle to entering the industry and the risk of failing during the Conformité Européenne (CE) marking process can be reduced. Theoretically, this work adds to the current discussions on the MDR by investigating its impact on business modeling and systematically incorporating it into a business model framework. In the literature on the MDR, the focus seems to be primarily on its challenges. Our work aims to develop solutions to tackle some of these challenges, thereby contributing to a rather unexplored research avenue.

The remainder of this work is structured as follows: the subsequent section provides background information on the MDR and clarifies related terms. The Methods section presents the research methodology, followed by the study’s results, including the derived conceptual framework. Finally, the key findings are discussed before we conclude the work.

### Theoretical Background

The World Health Organization defines *digital health* as the practice of using information and communication technology to meet health care needs [29]. Digital health includes concepts such as eHealth and emerging areas such as artificial intelligence [29]. Digital health technologies include SaMD [30]. In the EU, SaMD, which is not an in vitro medical device, is regulated under the MDR.

Aiming to increase the performance and safety of medical devices placed on the EU market [31], the MDR entered into force in 2021 [32], replacing the less stringent previous Medical Device Directive [33]. Multimedia Appendix 1 provides the definition of a medical device according to article 2 of the MDR. SaMD is software intended to be used for at least 1 medical purpose, performing these purposes without being part of a hardware medical device [10]. The software is intended to be used for medical purposes, such as diagnosing or treating diseases. The initial baseline for further steps in developing and approving medical devices is the determination of the intended purpose [34]. The intended purpose describes the designated use of the device as intended by the manufacturer [35]. For a device to qualify as a medical device, its intended purpose must be a medical purpose, in accordance with the definition of a medical device under the MDR (Multimedia Appendix 1).

A startup with a product that falls within the scope of SaMD in the EU needs to fulfill the safety and performance requirements specified by the MDR. These requirements include a QMS and technical documentation, among others [35]. The QMS captures processes and procedures to ensure the safety and performance of medical devices [31]. The technical documentation demonstrates their compliance with the defined safety and performance requirements [34]. Manufacturers are advised to apply harmonized standards and common specifications [35,36]. This helps prove compliance with the requirements and provides guidance on the necessary actions for the CE marking. Table 1 depicts the MDR’s key regulatory requirements and related standards relevant to SaMD startups. Notably, the standards mentioned in the table are not compulsory. However, they reflect the state of the art and are commonly used.

Although the requirements illustrated in Table 1 apply to all medical devices, the degree of detail is proportionate to the risk class. Medical devices can be classified into the following risk classes: I, IIa, IIb, and III [34]. The higher the risk class, the more stringent regulatory scrutiny is required. Compared to higher-risk classes, risk class I devices do not require a notified body for conformity assessment [13]. This saves the SaMD startup time and money. For risk classes III and implants, the MDR requires a clinical investigation [35] to demonstrate the safety and performance of the medical device. In addition, risk class III devices require a scrutiny procedure, increasing the regulatory burden. Thus, the risk class determines the regulatory burden for SaMD startups. It also affects the business model, for example, in financial terms, due to the required clinical investigation. Furthermore, it is significant to note that the requirements of the MDR are highly interdependent. For example, risk management and software development impact each other and must, therefore, be closely integrated to fulfill the safety and performance requirements of SaMD.

**Table 1:** Overview of key requirements regarding Regulation (EU) 2017/745, also known as the Medical Device Regulation (MDR), relevant for a software as a medical device startup.

Key requirement of the MDR	Description
QMS^a^	Encompasses elements such as quality planning, establishment of processes, and identification of resources [31] ISO^b^ 13485 is an international standard that formulates requirements for the QMS [37]
Technical documentation	Includes product-related information such as description and specification of the device, instructions for use, design, and manufacturing information [35]
Risk management	Includes evaluation of identified risks and the adoption of risk control measures to eliminate or reduce risks [35] ISO 14971 formulates requirements for risk management for medical devices [38]
Software life cycle	Software needs to be developed according to the state of the art [35] IEC^c^ 62304 reflects the state of the art for software development, from software planning to software release [39]
Clinical evaluation	Aims to generate, collect, examine, and evaluate clinical data to verify the safety and performance of a medical device [35] The basis for clinical evaluation is clinical data, which can be obtained from clinical investigations [35]
Usability engineering	Aims to identify and minimize use errors and thereby reduce risks associated with the use of the device [40] IEC 62366-1 reflects the state of the art in terms of usability engineering [40]
Cyber security management	MDR requires IT security measures [35] IEC 81001-5-1 provides guidance on integrating IT security throughout the software life cycle [41]
Postmarket surveillance	Covers activities after the medical device has been placed on the market Includes activities such as updating clinical evaluation and identifying options to improve usability [35]

^a^QMS: quality management system.

^b^International Organization for Standardization.

^c^IEC: International Electrotechnical Commission.

## Methods

### Overview

**Figure 1:**
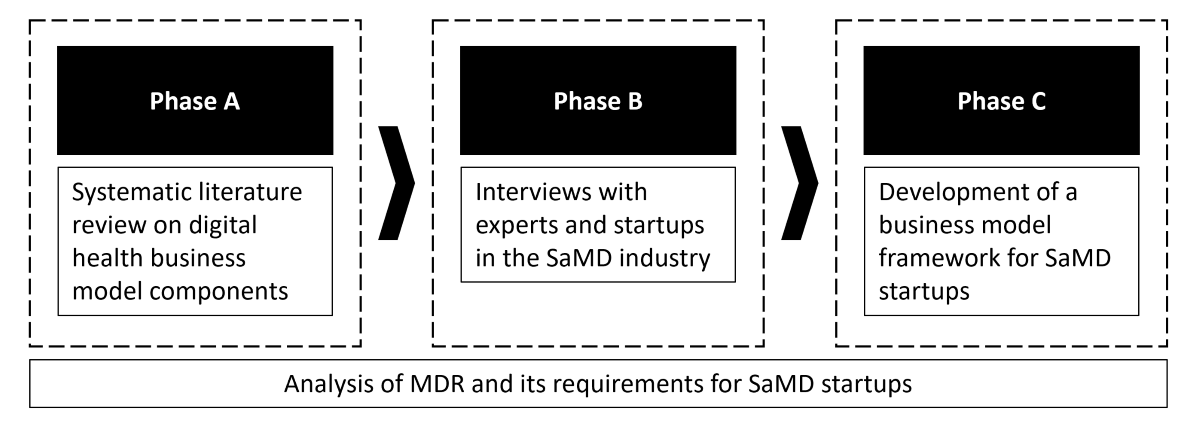
Research design of the business model framework for software as a medical device (SaMD) startups. MDR: Medical Device Regulation (Regulation (EU) 2017/745).

Our research design comprises 3 phases, as depicted in Figure 1. The basis for our study is the analysis of MDR requirements for SaMD. To understand MDR-related aspects, we mainly included the MDR itself [35], the standards mentioned in Table 1 [37-41], and guidance documents provided by the Medical Device Coordination Group. The guidance documents covered the documents on clinical investigation [42] and risk classification [43]. Additional sources included the works of Baumgartner et al [44] and Hastenteufel and Renaud [34].

Armed with the regulatory background information, we conducted a systematic literature review. We supplemented the results with qualitative data gathered through interviews with startups and experts in the EU SaMD industry. The input from the MDR, together with the results derived from the literature research and the expert interviews, served as the foundation for the development of the SaMD business model framework. An initial prototype was discussed within the research team, and feedback from potential users was gathered, which led to the framework presented in this work. Figure 1 depicts the overall research design of our study.

### Phase A: Systematic Literature Review

A systematic literature review is a structured and transparent approach to synthesizing research [45]. Kraus et al [46] define 4 steps of a systematic literature review, which were followed in this work: planning the review, identifying and evaluating studies, extracting and synthesizing data, and disseminating the review findings.

#### Planning the Review

The main goal of the literature review was to identify business model components used in the digital health sector. Because the literature does not explicitly address the topic of SaMD business model components, we broadened our search for digital health business model components.

#### Identifying Literature and Study Selection Strategy

**Figure 2:**
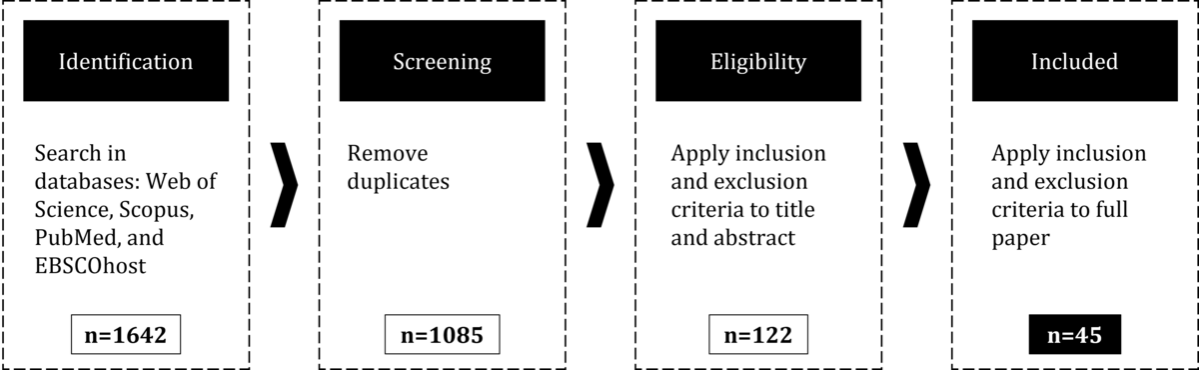
Overview of the systematic literature review on digital health business model components.

Figure 2 illustrates the study selection process. To identify relevant studies, we conducted a comprehensive search across four databases: Web of Science, Scopus, PubMed, and EBSCOhost. PubMed was selected because it covers medical topics. Web of Science and Scopus capture entrepreneurship literature. In EBSCOhost, the databases Academic Search Premier and Business Source Premier were chosen to account for academic and general business literature. The final search string encompasses the 2 core concepts of “digital health” and “business model,” along with related synonyms: (“digital health*” OR “electronic health*” OR ehealth* OR “e-health*” OR “mobile health*” OR mhealth* OR “m-health*” OR telehealth* OR “tele-health*” OR “connected health*” OR “connected-health*” OR medtech* OR “med-tech*” OR “medical technolog*” OR telemedic* OR “tele-medic*” OR “e-medic*” OR “electronic medic*” OR emedic* OR “digital medic*” OR “mobile medic*” OR “connected medic*”) AND (“Business Model*” OR “eBusiness Model*” OR “e-Business Model*”). After removing duplicates, we applied the inclusion and exclusion criteria to the title, abstract, and finally to the remaining full papers. Papers had to (1) be written in English, (2) be peer-reviewed articles, conference papers, or book chapters, and (3) mention business model components. Papers that presented a business model framework but did not specify the meaning of the components were excluded. This was done to avoid ambiguity and to prevent subsequent errors in the analysis. Furthermore, some papers developed business models for specific use cases. These models included components tailored to those cases and could not be generalized, making them irrelevant to our study. Papers identified in the databases that could not be accessed in full were also excluded from the study. The study selection process was conducted independently by 2 researchers. They discussed differences and reached a consensus to reduce selection bias. The systematic literature review resulted in 45 relevant papers [25,26,47-89] covering information about digital health business model components.

#### Extracting and Synthesizing Data

To synthesize the literature, we followed the approach by Webster and Watson [90] and developed a concept matrix. According to Webster and Watson [90], concepts define the organizational framework of a review. By reviewing the final set of papers, we gradually grouped business model components with similar meanings. To better understand the components, we looked for subcomponents and explanations for the respective constructs. Two researchers summarized the components with similar meanings and created definitions for the concepts. In case of discrepancies, 2 researchers discussed them to reach a consensus on the final concepts.

We assessed the quality of the studies using the Critical Appraisal Skills Program checklist for qualitative research [91]. It contains 10 questions with answer options including “yes,” “no,” and “can’t tell.” The quality of each study can be high (7-10), medium (4-6), or low (1-3). Two researchers independently filled out the checklist. Differences were discussed to reach a consensus. All studies scored 7 to 10, indicating high quality. The results are depicted in Multimedia Appendix 2 [25,26,47-89].

### Phase B: Expert Interviews

We conducted semistructured interviews to identify aspects of the business model that startups and experts in the EU SaMD industry perceived as important. The literature on business modeling for SaMD is a relatively new research avenue. Therefore, we had to expand the literature review to encompass literature on digital health in general. Interviews were crucial for our study to account for SaMD specifics. They were valuable in driving the development of the framework and enriched it with practical insights, including lessons learned and hints.

We searched for interviewees through web searches. Our focus was on SaMD startups in the EU and experts, such as startup coaches in incubators or accelerators in the SaMD industry. We aimed for a well-balanced mix of founders and industry experts. Potential interviewees were contacted via email or professional networking platforms. For the interviews, we followed a semistructured approach [92]. Semistructured interviews allow for flexibility in structure [92,93] and follow-up questions by the interviewer [94]. Therefore, complex topics can be explored, and an in-depth investigation of a topic is possible. Most interviews were conducted with chief executive officers and founder or cofounders of SaMD startups, which was ideally suited for our study and significantly augmented its value. The product range of the respective startups included various solutions, such as software to monitor and treat eye diseases or software for tracking skin conditions. In addition, opinions from experts with experience across a wide range of SaMD were included. An overview of the interviewees is provided in Multimedia Appendix 3. The interviews were recorded and transcribed. Subsequently, they were inductively coded following the approach by Gioia et al [95]. To ensure the objectivity of the coding, 2 researchers analyzed the transcripts and carried out the coding. Inconsistencies were discussed to derive common, aggregated dimensions.

### Ethical Considerations

The interviews were conducted in accordance with the relevant ethics guidelines and were exempt from undergoing an ethical review. All participants provided informed consent and agreed to the interview. Data collected from the interviews were deidentified to ensure confidentiality.

## Results

### Digital Health Business Model Components

The analysis of the final 45 papers demonstrated a substantial number of papers that developed business models based on the Business Model Canvas by Osterwalder and Pigneur [23], such as the papers by Bartels et al [47], Chen et al [48], and Christie et al [49]. Apart from the Business Model Canvas, the service, technology, organization, and finance (STOF) model by Bouwman et al [96] was applied to specific use cases [50,51]. Only a few authors derived their own framework [52,53]. Hwang and Christensen [52] developed a business model framework for health care, incorporating 4 components: value proposition, resources, processes, and profit formula. Lin et al [54] established a telemedicine framework and identified value proposition, partnership, resources, capability, service process, and cost structure as components. The remaining papers referred to other existing business model tools [97-102]. Multimedia Appendix 4 [25,26,47-89] provides an overview of the 45 papers, including their applied or derived business model frameworks.

Following the approach of Webster and Watson [90], we derived 14 business model components summarizing the constructs mentioned in the 45 papers. Multimedia Appendix 5 [25,26,47-89] depicts the concept matrix. Table 2 describes the 14 derived business model components. Of the 45 papers identified, only the study by Mueller [26] included regulation as a component, underlining its underrepresentation in the current digital health business model literature. The MDR, in particular, was not mentioned in any of the papers. Logically, no publication specifically focused on the business model of SaMD, underpinning the research gap. As shown in the concept matrix, value propositions and customer segments were frequently mentioned in the papers. This highlights their importance as key business model components.

The general business model components identified through the systematic literature review, on their own, do not sufficiently account for the specifics of the health care system. As Albert and Van der Auwermeulen [24] stated, the health care system is complex. Generic business model approaches, such as the Business Model Canvas, are inadequate to fully address its intricacies [24]. Therefore, to derive a business model framework tailored to SaMD startups, we enriched the results from the systematic literature review with expert interviews from the SaMD industry in the EU.

**Table 2:** Derived business model components from the systematic literature review and descriptions.

Business model component	Description
Customer segments	Determines different groups of people the company seeks to reach and serve [23].
Value propositions	Defines the products and services that provide value to customers [23].
Channels	Describes the communication, distribution, and sales channels used to deliver the value propositions to customers [23].
Customer relationships	Describes the type of relationship the company establishes with its customers [23].
Revenue model	Explains ways to make money, including revenue streams, revenue sources, and pricing strategies.
Core assets	Describes the key resources required to create value.
Core activities	Defines the central tasks to be carried out to create and provide value to the customer.
Cost structure	Describes the expenses associated with running the business [23].
Stakeholders	Groups or individuals that influence or are influenced by the fulfillment of an organization’s goals [103].
Digital solution	Describes the key characteristics and features of the SaMD^a^.
Key partners	Delineates the collaboration with individuals or organizations.
Capital and funding	Describes the capital and funding needed to cover costs as well as the analysis of funding options.
Market and competitors	Describes the market to be entered, including its competitors.
Regulation	Describes the regulations to be considered.

^a^SaMD: software as a medical device.

### Key Aspects of Business Modeling for SaMD Startups

Multimedia Appendix 6 depicts the inductive coding results of the interviews, according to Gioia et al [95]. The interviews made the MDR a focal aspect of SaMD business model (interviewee 10, interviewee 12, and interviewee 11). It was advised that the MDR should be considered from the beginning (interviewee 12). The interviews unequivocally demonstrated the impact of the MDR on other business model components, such as human resources, as part of core assets (interviewee 2 and interviewee 4). The MDR requires expertise that many startups lack. Therefore, ensuring the availability of human resources with the necessary knowledge to fulfill the requirements of MDR is crucial. In the interviews, the position of the quality manager was emphasized as essential (interviewee 6 and interviewee 4). It was recommended that a full-time position for a quality manager should be planned (interviewee 4). The MDR mandates the provision of “sufficient clinical evidence” [35]. However, the term *sufficient* is left open to interpretation, leading to ambiguity (interviewee 8). In such cases, startups often find it beneficial to seek advice from experienced consultants. Consultants can provide valuable insights specific to their case. In addition, the immense financial burden of the MDR was highlighted (interviewee 5 and interviewee 10). The CE marking process is lengthy (interviewee 8). During this period, financial sustainability must be maintained. Furthermore, clinical investigations have been mentioned as significant cost drivers (interviewee 5 and interviewee 8). With its limited resources, startups often turn to consulting companies seeking assistance with the MDR (interviewee 8). This adds to the already high overall costs associated with the CE marking. A financial analysis (interviewee 11), including follow-up funding (interviewee 4), seems essential. In addition, securing follow-up funding early (interviewee 4) was mentioned to avoid falling into a financial gap. Finding investors is challenging due to the long CE marking process and the resulting delayed revenue generation (interviewee 10). Investors need “some element of faith or guided imagination of where it can go” (interviewee 7). Therefore, it is essential to start the search process early on (interviewee 7).

Furthermore, the revenue model was emphasized as pivotal for the business model of SaMD startups (interviewee 8). Having the digital solution CE certified opens the door for additional revenue pathways, such as reimbursement by health insurance companies (interviewee 10). Because reimbursement systems are country specific, possible reimbursement pathways need to be considered for the target country (interviewee 10). Further revenue options are the self-payment market (interviewee 11), which is also country specific. For example, Germany was mentioned as a country with a low self-payment mentality (interviewee 10). On the contrary, Poland was named as one characterized by a relatively high self-payment mentality, where people are accustomed to spending money on their health care (interviewee 10). Thus, awareness of revenue sources and their potential in the target country is central to a viable revenue model.

Understanding the health care market was described as challenging (interviewee 6). Comprehending how the market works and identifying the biggest players and their relations were mentioned as significant obstacles for startups entering the health care sector (interviewee 6). The intricate stakeholder constellation was noted by several interviewees (interviewee 10). Understanding the interests of stakeholders, together with operational dynamics, was recognized as an obstacle (interviewee 10). In this regard, health insurance companies were highlighted as stakeholders whose functioning, interests, and expectations toward SaMD startups were difficult to grasp (interviewee 10). As a result, startups struggle to derive a value proposition for health insurance companies (interviewee 10). Because value propositions are to be considered for each customer segment from all angles (interviewee 11), the health insurance company needs to be addressed in case of a reimbursement revenue model.

Interviewee 9 highlighted the importance of competitor analysis. Distinct competitive advantages should be identified early (interviewee 9) to align the product accordingly. The complexity of the health care sector becomes evident once more when looking at customer segments. For SaMD startups, the user is often the patient, while the payer is the health insurance company (interviewee 6). This triangle of SaMD startup, health insurance company, and patient requires consideration. Interviewee 3 and interviewee 5 mentioned customer relationships, indicating the importance of reaching out to customers as early as possible to obtain feedback (interviewee 5). Interviewee 3 recommended creating a lock-in effect to bind the customer to the product. Key MDR-related activities mentioned were the QMS (interviewee 4) and the clinical evaluation (interviewee 5). The clinical evaluation was seen as unclearly formulated in the MDR. Terms such as “equivalent device” [24] were insufficiently described and left room for interpretation (interviewee 10). Apart from MDR-related activities, further key activities mentioned included research (interviewee 9) and the setup of a sales team (interviewee 5).

### Phase C: Development of the SaMD Business Model Framework

#### Overview

**Figure 3:**
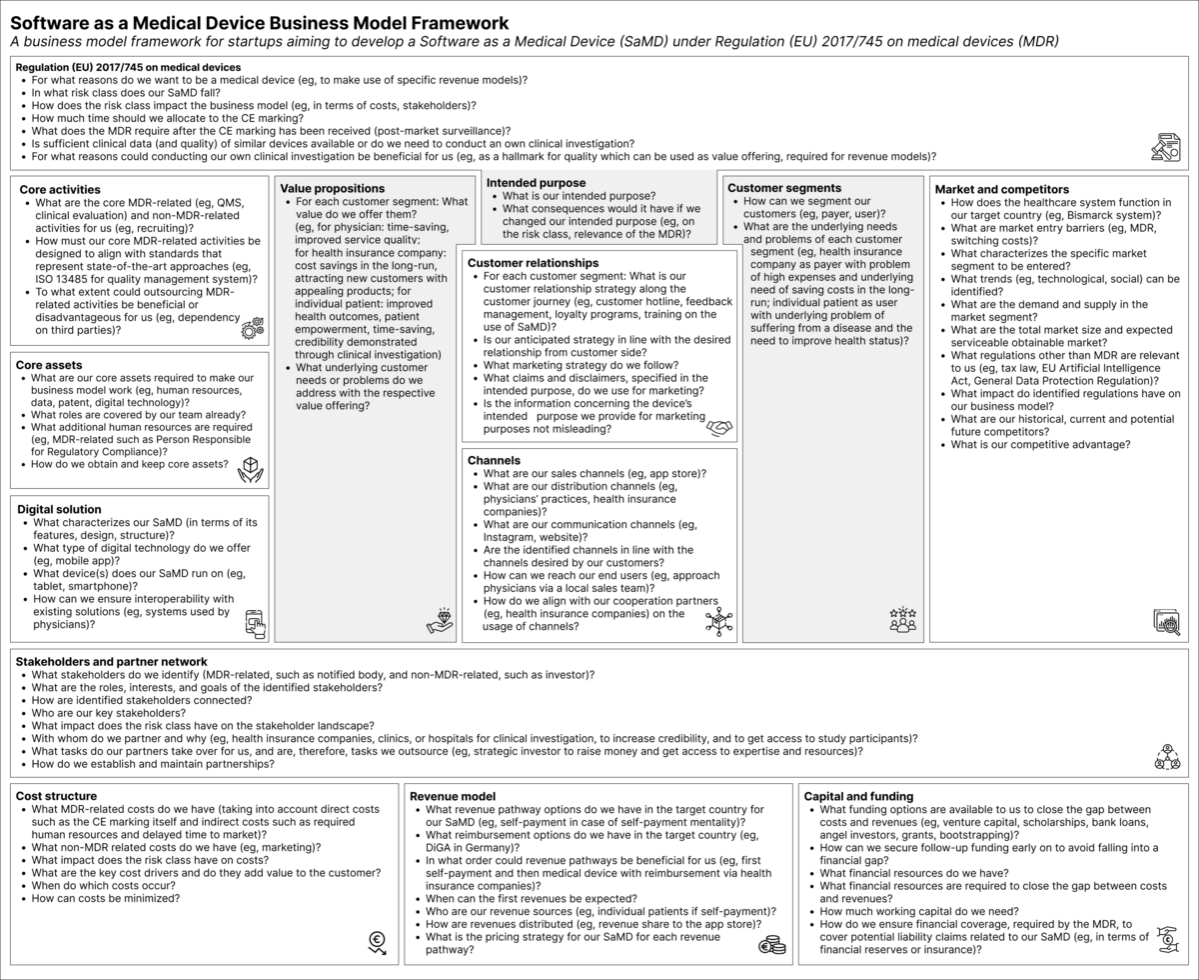
Software as a medical device business model framework.

In phase C (Figure 1), we developed the business model framework for startups aiming to develop SaMD, as shown in Figure 3 (for a higher-quality version of the figure, please see Multimedia Appendix 7). This was based on findings from the systematic literature review and expert interviews. It was further supplemented by MDR requirements, user feedback, and input from the research team on an initial prototype. In the following sections, we will initially address the visual layout of the framework. After that, the business model components of the SaMD business model framework will be discussed. This is followed by an analysis of the components’ interdependencies. Finally, the setting of the framework will be examined.

#### Visual Layout

The framework’s overall canvas-based structure resembles the Business Model Canvas by Osterwalder and Pigneur [23]. We consciously chose this representation because familiarity with it might simplify the use of our framework. The SaMD business model framework consists of 13 components. The MDR is positioned at the top of the framework, with the remaining components being arranged below. This placement indicates that the MDR requirements emanate from above, that is, the government, and are imposed on SaMD startups. Graphically opposed to the MDR is the financial part of the business model. It encompasses the components of *cost structure*, *revenue model,* and *capital and funding.* The *capital and funding* component aims to close the gap between costs and revenues. The financial part of the business model is the basis of business operations. Metaphorically, the financial basis can be seen as the legs of the framework, which enable it to function.

The centerpiece of the framework is the “regulatory value arc,” which is the trio consisting of *value propositions*, *intended purpose*, and *customer segments*. The dark gray color in Figure 3 highlights the importance of this trio. Changes in the regulatory value arc would imply changes to the remaining business model components. For instance, if customer needs change, the value offering might require adjustments to fit them. The intended purpose of the device should be adjusted according to the desired value offering. An adapted intended purpose can lead to a different risk class of the device, which could potentially require a clinical investigation. With the regulatory value arc being the centerpiece of the framework, radiating in all directions, the surrounding components need to be reconsidered in case of changes. The link between *value propositions* and *customer segments* is well known through the Value Proposition Canvas by Osterwalder et al [104]. The goal of the Value Proposition Canvas is to achieve a fit between customers on the one hand and the value offered by the product on the other hand [104]. We identified the *intended purpose* as a central aspect of the MDR in this relationship. The intended purpose can be seen as the connection between *value propositions* and *customer segments*. The intended purpose of the SaMD should reflect the value offering desired by the customer. Claims regarding the clinical and nonclinical benefits of the digital solution should include values specified in the value propositions. Once clinically validated, such claims may be used for marketing purposes to raise awareness of the SaMD. The value offering can be brought to customers through *channels* and *customer relationships*.

The framework has been visually designed so that its left side covers company-internal aspects of the business model, while its right side encompasses external elements. With *value propositions* being delivered from the company to *customer segments*, the internal and external sides are linked by the *intended purpose* from a regulatory perspective and by *channels* and *customer relationships* from a business perspective. *Digital solution*, *core assets*, and *core activities* are located on the company side, whereas *market and competitors* are placed on the external side. *Stakeholders and partner network* reflect both the internal and external sides. The stakeholder landscape in the SaMD industry has been described as complex and challenging to penetrate (interviewee 10). Furthermore, forming partnerships in the digital health sector is crucial (interviewee 6). The size of the component reflects its significance in the SaMD industry. Structurally, from top to bottom, *core activities* are positioned below the MDR. The requirements specified in the MDR directly cause a string of activities that contribute to developing the digital solution in conjunction with core assets.

#### Description of the Business Model Components

##### Regulation (EU) 2017/745 on Medical Devices

The interviews highlighted the MDR as a hurdle for startups aiming to develop SaMD business model (interviewee 10). Therefore, the MDR was added as a separate business model component to the components derived in Table 2 of the systematic literature review. The MDR component replaces the general regulation component. The main goal of the MDR component is to strategically consider the MDR in business modeling. Becoming aware of the key points of the MDR and its impact on the business model, not only from a financial but a comprehensive strategic perspective, is crucial for SaMD startups.

The intended purpose, the cornerstone of the MDR, comprises elements such as medical purposes and claims. The medical purpose lays the foundation for subsequent regulatory steps and should be carefully and tactically considered. Furthermore, the user and the use environment are central parts of the intended purpose, which need to be addressed deliberately. Frequently, claims related to the intended medical purpose are formulated. These claims can be used for marketing once CE marking is obtained, provided they are supported by clinical evidence.

##### Core Activities

Fulfilling the requirements of the MDR covers a significant part of the core activities of SaMD startups. Standards representing the state-of-the-art approaches can be used to implement the requirements specified in the MDR (Table 1). Apart from the QMS (interviewee 4), clinical evaluation is a central activity in the life cycle management of a medical device, along with software development. Risk management and the collection of customer feedback are ongoing activities during the CE marking process and after the launch of the SaMD. After the market launch, marketing and sales add to the postmarket surveillance activities. Activities can be outsourced. However, interviewee 8 recommended doing so with caution and building knowledge internally. Putting everything in the hands of external consultancies leaves startups at their mercy (interviewee 8). Startups need to engage with the MDR internally to be able to speak to consultancies at an equal level and ask qualified questions (interviewee 8).

##### Core Assets

Interviews and the literature highlighted human resources as a core asset (interviewee 5 and interviewee 4). The composition of the internal team is central to the success of SaMD startups (interviewee 6). Early on, it was recommended that the required qualifications and competencies be identified and compared with those existing in the internal team. If gaps are identified, additional human resources are hired or qualifications are outsourced. Key roles identified were the quality manager, responsible for the QMS (interviewee 6); the risk manager; the clinical investigator; and the person responsible for regulatory compliance. The role of the person responsible for regulatory compliance received particular consideration and is actively mentioned in the MDR [35].

##### Digital Solution

The *digital solution* component aims to describe the key aspects of the SaMD on a high level, primarily to facilitate the identification of the value offering. *Digital solution* includes the characteristics of the SaMD in terms of its features, design, and structure, as well as the type of digital technology and the identification of devices on which the SaMD is supposed to run.

##### Customer Segments

There are different ways of segmenting customers of SaMD startups. Possibly, the differentiation between user and payer is applicable (interviewee 6). In many cases, for SaMD startups, the payer is the health insurance company, and the user is the individual patient. Nevertheless, other options are possible, such as the self-payment market, with the user and payer being the same. Alternatively, the corporate employer could be the payer, and the employees the users of the SaMD. The needs and problems are to be determined for each identified customer segment to ensure a fit between customer needs and value offerings.

##### Value Propositions

To successfully capture the market for each customer segment, the SaMD must provide value. SaMD can provide value for individual patients by engaging them in self-treatment. Patients can be empowered, and their symptoms can be reduced. Although it seems challenging for SaMD startups to develop a value proposition for health insurance companies (interviewee 10), this is necessary if collaboration with health insurance companies is anticipated. In many cases, where there is a collaboration with the health insurance company, the latter is the payer of the product. Possible value offerings to health insurance companies are cost savings (interviewee 11) in the long term, attracting new customers with appealing products, and having satisfied customers who stay with the health insurance company. In addition, health insurance companies often require a clinical investigation that clinically proves the device’s safety and performance. Typical value offerings to physicians are time saving, additional income, and improved diagnosis or therapy.

##### Customer Relationships

A customer relationship strategy needs to be worked out for each customer segment [15]. Potential customer relationships can be established and strengthened via customer hotlines, feedback management, or loyalty programs. Training on using the SaMD, for instance, at a physician’s location, can also nurture the customer relationship. Interviewee 3 recommended a lock-in effect for customer retention. Marketing is essential to customer relationship management and should be considered early on. Once the CE marking has been obtained, the SaMD does not sell itself; it needs to be advertised. For advertisement, the MDR prohibits providing misleading information concerning the device’s intended purpose. This prohibition includes suggesting uses for the device that are not specified in the intended purpose for the conformity assessment [24]. Since all claims made in the intended purpose must be supported by clinical evidence, only those that are clinically validated may be used for marketing purposes on the website, social media platforms, and other marketing platforms.

##### Channels

*Channels* encompasses sales, distribution, and communication channels [23]. The channel type depends on the customer segment, customer needs and preferences, and the selected revenue model. Because many SaMDs are apps, common sales channels are app stores, such as Google Play Store and Apple App Store. In a reimbursement model with a health insurance company, the health insurance company can act as a distribution channel by sending activation codes for downloading the SaMD to the end user. Because physicians can prescribe SaMDs, such as digital health applications (DiGAs) in Germany, they can also be part of the distribution channel. Common communication channels for SaMD startups are the website or the health insurance company that promotes SaMD via their existing channels.

##### Market and Competitors

The health care market is described as being complex [105]. Understanding how it functions is crucial before entering it. The target country’s market analysis should include an investigation of the established health insurance system. In addition, the target market segment needs to be thoroughly inspected. This inspection should include reviewing existing solutions and trends in the market segment, as well as determining the expected serviceable obtainable market. Notably, analyzing competitors is essential for both understanding the target market segment and developing product differentiation strategies. For the competitor analysis, not only current but also historic and potential future competitors should be investigated.

The MDR has been perceived as a regulatory challenge for SaMD startups when developing SaMD (interviewee 10). Nevertheless, further regulations that might be specific to markets or products should be considered and checked for relevance. These regulations include, for example, the General Data Protection Regulation, the EU Artificial Intelligence Act, and country-specific regulations.

##### Stakeholders and Partner Network

Although the health care ecosystem is difficult to navigate, it is fundamental to identify its actors, their roles, and interests. Understanding the relationship between stakeholders is crucial for developing effective stakeholder management. In the interviews, health insurance companies were described as stakeholders that are difficult to understand (interviewee 10). Nevertheless, they are crucial to analyze. Once stakeholders have been analyzed for their roles and interests, their handling needs to be determined. Stakeholder management can vary between SaMD startups. For example, interviewee 11 mentioned their early collaboration with health insurance companies.

Moreover, notified bodies are essential stakeholders for all medical devices with a risk class higher than risk class I. Given the long waiting times (interviewee 10) in some countries, identifying potential notified bodies early is crucial.

The interviews highlighted the importance of having partners (interviewee 6). A partner network is crucial, especially in the digital health sector (interviewee 6). Potential partners could be local sales teams, health insurance companies, strategic investors, or clinics involved in the clinical investigation. Research institutions, pharmaceutical companies, and medical technology companies should also be considered possible collaborators. Partners can be essential stakeholders in accessing expertise and resources, such as patients, for clinical investigation and to increase credibility.

##### Cost Structure

The CE marking process is costly, especially for startups, which often lack financial resources. The required human resources represent a significant cost driver, including, for example, external consultants for support and, if necessary, the involvement of a notified body. Furthermore, the clinical investigation can be expensive. For SaMD startups, awareness of the additional investments incurred in conjunction with the CE marking is essential. Even after the CE marking has been obtained, costs will not diminish. With the postmarket surveillance activities, fulfilling the requirements of the MDR remains a significant cost driver. In addition, costs for marketing and sales are added. Interviewee 11 emphasized the considerable costs of building a sales team.

##### Revenue Model

The CE marking opens the door for new revenue models. The CE marking is not necessarily required for products in the self-payment market, provided that the product can be legitimately positioned in a way that it does not qualify as a medical device. Nevertheless, the decision to forgo the CE marking should be made carefully, as the CE marking is often perceived as a hallmark of quality and trust. For reimbursement with health insurance companies, the CE marking and a clinical investigation are often necessary for collaboration. The specific reimbursement options are specific to countries and products. In Germany, for example, DiGA is a possible reimbursement option for SaMDs that aim to get reimbursed by all statutory health insurance companies. To become a DiGA, the SaMD needs to fulfill additional national requirements.

##### Capital and Funding

Due to the delayed revenues and high expense levels, capital and funding should be well planned. Because small businesses often lack financial resources [106], funding options, such as private equity and debt financing, should be considered. Funding options should be examined early on. Obtaining the CE marking is a lengthy process that delays revenue generation, making it difficult to identify investors (interviewee 10). Once funding is secured, follow-up funding options should be considered early on (interviewee 4) to avoid falling into a financial gap.

##### Interdependencies

**Figure 4:**
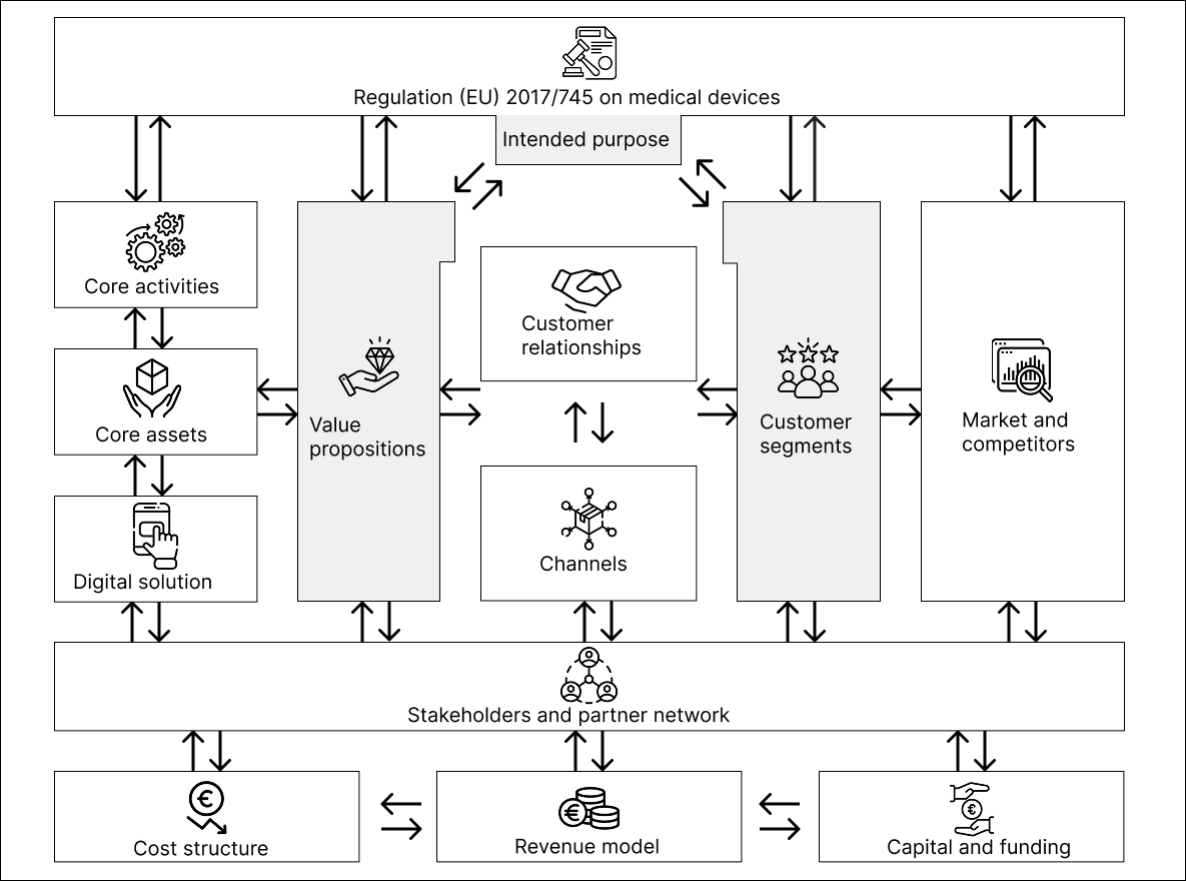
Interdependencies of the software as a medical device business model components.

Figure 4 depicts the interdependencies between each of the business model components. As shown in the figure, changing 1 component of the SaMD business model framework impacts the remaining components directly or indirectly via other components. For example, if *customer segments* change, likely *value propositions* will change due to the resulting alterations in the needs and preferences of the adapted customer segments. *Customer relationships* and *channels* are specific to each customer segment and need to be revised accordingly. With a changed value offering, the *digital solution* needs to be reconsidered, and therefore, *core assets* and *core activities* necessary to derive the value offering need to be reconsidered as well. An adapted SaMD translates into modified *stakeholders and partner network* and adjusted costs necessary to develop the solution. Revenues also depend on the type of digital solution, and funding, in turn, depends on costs and revenues. An adapted digital solution requires a modified market and competitor analysis. Likewise, an adapted digital solution might imply changes in the intended purpose, thereby requiring an adjustment of the risk class. In summary, the business model components of the SaMD business model framework are interdependent. It is noteworthy that the requirements, as listed in Table 1, remain consistent for each SaMD. However, what might change is the extent and precise implementation of the requirements, which depend on the risk class of the SaMD and are therefore driven by its intended purpose.

##### Setting

The SaMD business model framework targets startups aiming to develop SaMD under the MDR. Startups experience different development phases. We followed the 4 development stages applied in the work by Santisteban and Mauricio [107]: seed, early, growth, and expansion. In the seed stage, startups usually have a first product idea in mind and develop an initial draft of a business model. With a rough software product idea in the digital health sector in mind, startups assess whether their product falls under the MDR and fulfills the criteria of SaMD. In such a scenario, where the product fulfills the criteria of SaMD, startups begin using the SaMD business model framework. It assists in ensuring that all critical aspects of business modeling are considered early on while directly strategically integrating the central aspects of the MDR on a high level. Instead of being caught up in the intricate details of the MDR requirements, the framework provides a holistic picture and aims to strategically consider the MDR in business modeling. Often, startups in the seed and early stages lack regulatory expertise and are overwhelmed by the requirements of the MDR. The framework, with its strategically crafted guiding questions, supports SaMD startups in navigating the MDR. An additional use case of the framework is when startups decide which revenue model to choose. With a product idea in mind, the question of how to possibly earn money with it arises. For reimbursement by health insurance companies, such as through DiGA, the software must be SaMD and fulfill the requirements of the MDR. In these instances, the framework unfolds its strength. It showcases what the business model would look like, considering the MDR. Instead of focusing on financial implications, the framework provides a holistic picture of integrating the MDR in business modeling. Multimedia Appendix 8 shows an example filled-out version of the framework.

Business models are not one-time concepts [108]. Instead, they are derived iteratively over time and are subject to change. Especially in highly regulated sectors, such as the SaMD sector, startups need to remain agile, stay updated with regulatory developments, and adjust their business models as needed. This need for adaptability includes potential changes to the MDR and upcoming relevant regulations, such as the EU Artificial Intelligence Act.

## Discussion

### Summary

The MDR is a complex and challenging topic, especially for startups with limited resources. As the expert interviews demonstrated, the impact of the MDR on business modeling for startups aiming to obtain the CE marking for their SaMD is vast; however, it is underresearched, as the systematic literature review has illustrated. Therefore, the objective of this study was to understand the relationship between MDR and business modeling and to derive a business model framework that supports SaMD startups in developing a business model. To achieve this goal, we followed a 3-step approach. First, we conducted a systematic literature review to identify business model components relevant to the digital health sector. In total, 45 papers were identified. The review highlighted the lack of analysis of business models specific to SaMD startups. To make our conceptual framework tailored to SaMD startups, we conducted, in a second step, 13 expert interviews with specialists in the EU SaMD industry. We enriched the results with feedback from potential users and the research team on an initial prototype. Furthermore, we supplemented the results with the requirements of the MDR, including related literature and state-of-the-art standards. On the basis of this input, we conceptualized the SaMD business model framework depicted in this work.

### Principal Findings

The interviews and the literature unveiled that existing business model frameworks, such as the Business Model Canvas by Osterwalder and Pigneur [23], reach their limits regarding industry-specific cases. SaMD startups are embedded in a complex digital health care system with multiple stakeholders and a tricky customer constellation. The situation of SaMD startups is challenging due to many factors, and implementing the MDR in addition to these factors makes the challenge even greater. For business modeling, the MDR can be seen as a driving force impacting the entire business model, while being a pivotal component itself. Therefore, the SaMD business model framework is designed from a startup’s perspective, with the aim of developing SaMD business model. The MDR is considered in each derived business model component, facilitating business modeling for SaMD startups. In addition, the visual layout of the framework delineates the relation between business model components and demonstrates how the MDR fits into this constellation.

The MDR is not the only regulation that SaMD startups need to consider. Country-specific laws on marketing, taxes, or labor, as well as EU-wide regulations, such as the General Data Protection Regulation, need to be considered. Undoubtedly, these regulations have a ripple effect on business modeling as well. However, among all these regulations, the MDR seems to hold a special position because of its complexity, setting a series of consequential tasks. Evidently, in the interviews with SaMD startups and experts in this industry, the MDR has been highlighted as a hurdle in business modeling (interviewee 10 and interviewee 11). Furthermore, the regulatory value arc depicted in Figure 3 illustrates the close interrelation between the *intended purpose*, *value propositions*, and *customer segments*, underlining the embeddedness of the MDR in the business model of SaMD startups. Consequently, the MDR’s pivotal role needs to be directly integrated into the business modeling of SaMD startups.

Furthermore, we want to mention the identified regulatory value arc as another principal finding of our study. The trio of *intended purpose*, *value propositions*, and *customer segments* is the centerpiece of the framework and warrants particular attention. Although the link between *value propositions* and *customer segments* is well known, we identified the notable impact of the MDR on this duo, highlighting the extensive role of the MDR in business modeling. The intended purpose describes the designated use of the device as intended by the manufacturer and needs to cover at least 1 medical purpose to qualify as a medical device [35]. The user, use environment, potential contraindications, and claims, along with possible disclaimers, are formulated apart from the medical purpose. The description of the users and the link to customer segments are evident. Besides, claims are statements that can be used to advertise SaMD. Claims should reflect the value offering desired by the customer to ensure customer needs are met with the SaMD. Therefore, the intended purpose, as the cornerstone of the MDR, is the pivotal element bridging value propositions and customer segments, forming the trio “regulatory value arc”*.* With the intended purpose, a connection between the company-internal side of the business model framework and the external side of the framework is established. Claims formed in the context of the intended purpose are carried out via channels and customer relationships from the company to the customers. Thus, for a startup aiming to develop SaMD, it is imperative to consider the regulatory value arc.

In the interviews and the literature, the clinical investigation was mentioned as a significant hurdle in the context of the MDR (interviewee 10). Studies are expensive and time consuming. They slow down the launch of the SaMD on the market. Data from clinical investigations are mandatory for risk class III and implantable medical devices [109]. For the remaining risk classes, a clinical investigation does not need to be undertaken if sufficient clinical data for performing the clinical evaluation can be obtained from literature or other sources from equivalent devices. Following this line of reasoning, innovative products are unlikely to refer to existing clinical data, requiring them to conduct their own clinical investigations. In that sense, startups with innovative products have ironically a disadvantage with the MDR compared to less innovative products. For less innovative products, clinical data are often already on the market.

Each clinical benefit claim included in the intended purpose needs clinical evidence. Reflecting on the challenges of the clinical investigation mentioned earlier, it is worth trying to circumvent the clinical investigation by adapting the intended purpose. This consideration must be thoroughly evaluated. Possible advantages include cost and time savings, while potential disadvantages may involve an adjusted product focus and challenges in collaborating with health insurance companies.

### Theoretical and Practical Implications

Our work has several theoretical and practical contributions. From a theoretical perspective, our work contributes to both digital health literature and business model literature. Although the literature on business models is vast, the impact of regulations on business modeling has not been sufficiently investigated. Business model frameworks, such as the Business Model Canvas by Osterwalder and Pigneur [23], do not explicitly include regulation in the framework. This can be fatal when regulations such as the MDR occupy a pivotal role that significantly impacts the business modeling process. In our work, we investigated the impact of the MDR on business modeling for SaMD startups. Our work clearly showed that the MDR plays a central role in the business model of SaMD startups and needs to be considered from the beginning. This study lays a foundation for a research stream examining business modeling for SaMD startups specifically and the relationship between regulations and business models. Furthermore, the literature on SaMD startups mainly focuses on their challenges, especially in the context of the MDR. Concrete solutions for how these challenges can be tackled are missing. Our work provides a way to address some of these hurdles. This work lays the groundwork for a solution-oriented approach to tackling SaMD startups’ challenges by providing a SaMD business model framework.

From a practical perspective, the derived SaMD business model framework provides a hands-on and all-in-one solution for business modeling. The framework enables the description and design of a business model for SaMD startups. The medical device industry is complex and is associated with hurdles for startups. Maintaining oversight in this complex setting is challenging. The framework can reduce complexity and minimize the risk of overlooking key aspects. It covers all factors relevant to developing a business model for SaMD. From the perspective of SaMD startups, each business model component directly incorporates MDR-specific information. Furthermore, with the visualization of the framework, an overall picture of business modeling for SaMD startups becomes evident. Business model frameworks provide a level of customizable abstraction that can be adjusted to fit the specific use case [110]. They provide a common language [110] supporting mutual understanding within the startup and its partner network. Moreover, business model frameworks are often used for pitching to investors. This framework can assist in pitching in front of them. In this way, the issue of attracting suitable investors in the SaMD industry is addressed in this work.

Ideologically, the concepts of startups and MDR seem to be colliding [14]. Startups, often young, dynamic, and packed with innovative ideas, see themselves confronted with a strict, documentary-rich MDR, which slows down innovation. Startups often lack regulatory experience and have limited resources, whereas the MDR requires expertise and substantial financial and human resources. The SaMD business model framework aims to bring these worlds together for the benefit of both parties. The framework assists startups in integrating the seemingly opposite MDR world into their daily business.

The framework has been derived based on the needs of SaMD startups and, therefore, provides a tailored, practical solution to them. Beyond stand-alone SaMDs, hybrid models involving software and hardware are possible. Combining hardware with software makes it necessary to clarify whether hardware, software, or both fall under the category of medical devices. In addition, with a hardware component, the MDR requires the consideration of specific hardware standards, such as the International Electrotechnical Commission (IEC) 60601-1 on medical electrical equipment. Therefore, core activities in a hybrid model differ from pure stand-alone software. For example, hardware production must be considered, resulting in higher investments compared to stand-alone software. Distribution to customers is different for hybrid solutions as well. The derived SaMD business model framework, in its basic structure, can be applied to hybrid models that combine software and hardware. However, the guiding questions do not entirely align with the needs of startups working with hybrid models and would require adjustments.

### Limitations and Future Research

This work is not without limitations. First, our work focused only on the EU region. The framework might not be applicable for startups aiming to bring SaMD to the market in non-EU countries. This is due to differences in the setting, such as a distinct regulatory environment. Second, our research focused on stand-alone software. Therefore, we did not incorporate cases that include hardware. Future research could adapt the derived framework to be tailored to hardware medical devices or embedded software. Third, our work focused on Regulation (EU) 2017/745. Regulation (EU) 2017/746 on in vitro diagnostic medical devices was not targeted. Fourth, the digital health sector is fast paced and changes rapidly. Our work provides a snapshot of the current setting for SaMD startups in the EU. Finally, the framework presented in this work has not been evaluated yet. Apart from experts in the EU SaMD industry, seed and early-stage SaMD startups, as the target users of the framework, should apply it in practice to validate its usefulness. The framework could, for example, be shared with accelerators, where startups develop the business model for their SaMD. An observation protocol could gather insights into how startups work with the framework. In addition, these startups could be interviewed to evaluate the framework based on predefined criteria. In addition, interviews could be conducted with startups that are in a later stage. These SaMD startups have already developed a business model for their SaMD and can offer valuable insights and feedback based on their experience. The validation of the framework could be a potential avenue for future research.

### Conclusions

The field of SaMD is rapidly evolving and offers various opportunities. In our work, we analyzed business modeling in the EU SaMD industry, focusing on startups as a driving force in this industry. Initially, we conducted a systematic literature review and derived a concept matrix. The work was then supplemented with expert interviews. In addition, the requirements of the MDR relevant to SaMD startups and related state-of-the-art standards were considered to develop the SaMD business model framework. Our study is a first step toward mitigating the challenging situation of SaMD startups. Our contributions offer valuable insights into the relationship between MDR and business modeling. The work provides a hands-on solution for startups aiming to develop SaMD under the MDR. Our study could assist SaMD startups in their business modeling process and reduce complexity.

## Appendix

Multimedia Appendix 1 Definition of a medical device according to regulation 2017/745 on medical devices.

Multimedia Appendix 2 Critical appraisal skills program checklist.

Multimedia Appendix 3 Sample of experts interviewed.

Multimedia Appendix 4 Overview of final papers from the systematic literature review, including the mentioned business model frameworks applied or derived.

Multimedia Appendix 5 Concept matrix based on the systematic literature review.

Multimedia Appendix 6 Overview of inductive coding according to Gioia et al [52].

Multimedia Appendix 7 Software as a medical device business model framework.

Multimedia Appendix 8 Example of filled-out software as a medical device business model framework.

## Abbreviations

CE: Conformité Européenne
DiGA: digital health application
EU: European Union
IEC: International Electrotechnical Commission
MDR: Medical Device Regulation
QMS: quality management system
RQ: research question
SaMD: software as a medical device
STOF model: service, technology, organization, and finance model
