# Supplementary material for: A Business Model Framework for Software as a Medical Device Startups in the European Union: Mixed Methods Study
Source: J Med Internet Res. 2025 May 23;27:e67328. doi: 10.2196/67328 (PMC12144475; doi:10.2196/67328)
Supplement: Multimedia Appendix 1 [file jmir_v27i1e67328_app1.docx]

Multimedia Appendix 1. Definition *medical device* according to article 2(1) of regulation (EU) 2017/745 on medical devices.

“‘medical device’ means any instrument, apparatus, appliance, software, implant, reagent, material or other article intended by the manufacturer to be used, alone or in combination, for human beings for one or more of the following specific medical purposes:

— diagnosis, prevention, monitoring, prediction, prognosis, treatment or alleviation of disease,

— diagnosis, monitoring, treatment, alleviation of, or compensation for, an injury or disability,

— investigation, replacement or modification of the anatomy or of a physiological or pathological process or state,

— providing information by means of in vitro examination of specimens derived from the human body, including organ, blood and tissue donations,

and which does not achieve its principal intended action by pharmacological, immunological or metabolic means, in or on the human body, but which may be assisted in its function by such means.

The following products shall also be deemed to be medical devices:

— devices for the control or support of conception;

— products specifically intended for the cleaning, disinfection or sterilisation of devices as referred to in Article 1(4) and of those referred to in the first paragraph of this point.” [1]

**References**

[1] Regulation (EU) 2017/745 of the European Parliament and of the Council of 5 April 2017 on medical devices, amending Directive 2001/83/EC, Regulation (EC) No 178/2002 and Regulation (EC) No 1223/2009 and repealing Council Directives 90/385/EEC and 93/42/EEC (Text with EEA relevance. ). European Union. URL: https://eur-lex.europa.eu/legal content/EN/TXT/?uri=CELEX%3A32017R0745 [accessed 2024-02-15]
