# Supplementary material for: A Business Model Framework for Software as a Medical Device Startups in the European Union: Mixed Methods Study
Source: J Med Internet Res. 2025 May 23;27:e67328. doi: 10.2196/67328 (PMC12144475; doi:10.2196/67328)
Supplement: Multimedia Appendix 2 [file jmir_v27i1e67328_app2.docx]

Multimedia Appendix 2. Critical Appraisal Skills Programme checklist.

| Classification  of Quality | Score | Item 10 | Item 9 | Item 8 | Item 7 | Item 6 | Item 5 | Item 4 | Item 3 | Item 2 | Item 1 | Author (Year) |
| --- | --- | --- | --- | --- | --- | --- | --- | --- | --- | --- | --- | --- |
| High | 7 | Y | Y | Y | Can’t tell | Can’t tell | Y | Can’t tell | Y | Y | Y | Acheampong and Vimarlund (2015) [1] |
| High | 7 | Y | Y | Y | Can't  tell | Can't tell | Y | Can’t tell | Y | Y | Y | Alnahdi (2023) [2] |
| High | 10 | Y | Y | Y | Y | Y | Y | Y | Y | Y | Y | Bartels et al. (2022) [3] |
| High | 7 | Y | Y | Y | Can't  tell | Can't  tell | Y | Can’t tell | Y | Y | Y | Chen et al. (2013) [4] |
| High | 10 | Y | Y | Y | Y | Y | Y | Y | Y | Y | Y | Christie et al. (2020) [5] |
| High | 7 | Y | Y | Y | Can't  tell | Can't  tell | Y | Can’t tell | Y | Y | Y | Fachinger and Schöpke (2014) [6] |
| High | 7 | Y | Y | Y | Can’t  tell | Can’t  tell | Y | Can’t tell | Y | Y | Y | Fife and Pereira (2011) [7] |
| High | 10 | Y | Y | Y | Y | Y | Y | Y | Y | Y | Y | Fusco and Turchetti (2015) [8] |
| High | 7 | Y | Y | Y | Can’t tell | Can’t tell | Y | Can’t tell | Y | Y | Y | García-Holgado et al. (2019) [9] |
| High | 8 | Y | Y | Y | Y | Can’t tell | Y | Can’t tell | Y | Y | Y | Grustam et al. (2017) [10] |
| High | 7 | Y | Y | Y | Can’t tell | Can’t tell | Y | Can’t tell | Y | Y | Y | Grustam et al. (2018) [11] |
| High | 7 | Y | Y | Y | Can’t tell | Can’t tell | Y | Can’t tell | Y | Y | Y | Hwang and Christensen (2008) [12] |
| High | 7 | Y | Y | Y | Can’t tell | Can’t tell | Y | Can’t tell | Y | Y | Y | Kho et al. (2020) [13] |
| High | 8 | Y | Y | Y | Can’t tell | Can’t tell | Y | Y | Y | Y | Y | Kijl et al. (2010) [14] |
| High | 7 | Y | Y | Y | Can’t tell | Can’t tell | Y | Can’t tell | Y | Y | Y | Kimble (2015) [15] |
| High | 10 | Y | Y | Y | Y | Y | Y | Y | Y | Y | Y | Korsgaard et al. (2021) [16] |
| High | 7 | Y | Y | Y | Can’t tell | Can’t tell | Y | Can’t tell | Y | Y | Y | Kriegel et al. (2017) [17] |
| High | 7 | Y | Y | Y | Can’t  Tell | Can’t tell | Y | Can’t tell | Y | Y | Y | Lee and Chang (2016) [18] |
| High | 7 | Y | Y | Y | Can’t tell | Can’t tell | Y | Can’t tell | Y | Y | Y | León et al. (2016) [19] |
| High | 7 | Y | Y | Y | Can’t tell | Can’t tell | Y | Can’t tell | Y | Y | Y | Lin et al. (2010) [20] |
| High | 9 | Y | Y | Y | Can’t  tell | Y | Y | Y | Y | Y | Y | Lin et al. (2011) [21] |
| High | 8 | Y | Y | Y | Can’t  tell | Can’t tell | Y | Y | Y | Y | Y | Martin et al. (2018) [22] |
| High | 8 | Y | Y | Y | Can’t  tell | Can’t tell | Y | Y | Y | Y | Y | Mueller (2019) [23] |
| High | 7 | Y | Y | Y | Can’t  tell | Can’t  tell | Y | Can’t tell | Y | Y | Y | Nikou and Bouwman (2017) [24] |
| High | 10 | Y | Y | Y | Y | Y | Y | Y | Y | Y | Y | Oderanti and Li (2018) [25] |
| High | 10 | Y | Y | Y | Y | Y | Y | Y | Y | Y | Y | Oderanti et al. (2021) [26] |
| High | 7 | Y | Y | Y | Can’t tell | Can’t tell | Y | Can’t tell | Y | Y | Y | Pascarelli et al. (2023) [27] |

| Classification of Quality | Score | Item 10 | Item 9 | Item 8 | Item 7 | Item 6 | Item 5 | Item 4 | Item 3 | Item 2 | Item 1 | Author (Year) |
| --- | --- | --- | --- | --- | --- | --- | --- | --- | --- | --- | --- | --- |
| High | 7 | Y | Y | Y | Can’t  tell | Can’t  tell | Y | Can’t tell | Y | Y | Y | Pereira et al. (2016) [28] |
| High | 9 | Y | Y | Y | Can’t tell | Y | Y | Y | Y | Y | Y | Peters et al. (2015) [29] |
| High | 7 | Y | Y | Y | Can’t tell | Can’t tell | Y | Can’t tell | Y | Y | Y | Pistorio et al. (2017) [30] |
| High | 7 | Y | Y | Y | Can’t  tell | Can’t  tell | Y | Can’t tell | Y | Y | Y | Radić et al. (2022) [31] |
| High | 7 | Y | Y | Y | Can’t  tell | Can’t tell | Y | Can’t tell | Y | Y | Y | Schuhmacher et al. (2023) [32] |
| High | 10 | Y | Y | Y | Y | Y | Y | Y | Y | Y | Y | Sprenger (2016) [33] |
| High | 10 | Y | Y | Y | Y | Y | Y | Y | Y | Y | Y | Steinhauser (2019) [34] |
| High | 10 | Y | Y | Y | Y | Y | Y | Y | Y | Y | Y | Sterling and LeRouge (2019) [35] |
| High | 7 | Y | Y | Y | Can’t  tell | Can’t tell | Y | Can’t tell | Y | Y | Y | Tageo et al. (2020) [36] |
| High | 10 | Y | Y | Y | Y | Y | Y | Y | Y | Y | Y | Thamjamrassri et al. (2018) [37] |
| High | 7 | Y | Y | Y | Can’t tell | Can’t tell | Y | Can’t tell | Y | Y | Y | van Limburg et al. (2015) [38] |
| High | 9 | Y | Y | Y | Can’t tell | Y | Y | Y | Y | Y | Y | van Meeuwen et al. (2015) [39] |
| High | 10 | Y | Y | Y | Y | Y | Y | Y | Y | Y | Y | Velayati et al. (2021) [40] |
| High | 10 | Y | Y | Y | Y | Y | Y | Y | Y | Y | Y | Velayati et al. (2022) [41] |
| High | 8 | Y | Y | Y | Y | Can’t tell | Y | Can’t tell | Y | Y | Y | Velayati et al. (2022) [42] |
| High | 10 | Y | Y | Y | Y | Y | Y | Y | Y | Y | Y | Veld et al. (2011) [43] |
| High | 9 | Y | Y | Y | Can’t tell | Y | Y | Y | Y | Y | Y | Visser et al. (2010) [44] |
| High | 7 | Y | Y | Y | Can’t tell | Can’t tell | Y | Can’t tell | Y | Y | Y | Viswanadham (2021) [45] |

Abbreviations: Y = Yes; N = No; Item 1 = Clear statement of aim; Item 2 = Appropriate qualitative methodology; Item 3 = Appropriate research design; Item 4 = Appropriate recruitment strategy; Item 5 = Adequacy in data collection; Item 6 = Adequate consideration of relationship between researcher and participants; Item 7 = Ethical considerations; Item 8 = Rigor in data analysis; Item 9 = Clear statement of findings; Item 10 = Overall value of research.

**References**

[1] Acheampong F, Vimarlund V. Business models for telemedicine services: a literature review. Health Systems. 2015;4(3):189–203. [doi: 10.1057/hs.2014.20]

[2] Alnahdi S. An psychological mindset based stress free business model for digital health? Journal for Re Attach Therapy and Developmental Diversities. 2023;6(4s):401-412.

[3] Bartels SL, Johnsson SI, Boersma K, Flink I, McCracken LM, Petersson S, et al. Development, evaluation and implementation of a digital behavioural health treatment for chronic pain: study protocol of the multiphase DAHLIA project. BMJ Open. 2022;12(4):e059152. [doi: 10.1136/bmjopen-2021-059152]

[4] Chen S, Cheng A, Mehta K. A review of telemedicine business models. Telemedicine and e-health. 2013;19(4):287–297. [doi: 10.1089/tmj.2012.0172]

[5] Christie HL, Boots LMM, Peetoom K, Tange HJ, Verhey FRJ, De Vugt ME. Developing a plan for the sustainable implementation of an electronic health intervention (partner in balance) to support caregivers of people with dementia: case study. JMIR Aging. 2020;3(1):e18624. [doi: 10.2196/18624]

[6] Fachinger U, Schöpke B. Business model for sensor-based fall recognition systems. Informatics for Health and Social Care. 2014;39(3–4):305–318. [doi: 10.3109/17538157.2014.931855]

[7] Fife E, Pereira F. Digital home health and mHealth: prospects and challenges for adoption in the U.S. 2011 50th FITCE Congress - ‘ICT: Bridging an Ever Shifting Digital Divide’; Aug 31 – Sept 03, 2011; Palermo, Italy. [doi: 10.1109/FITCE.2011.6133431]

[8] Fusco F, Turchetti G. Interactive business models for telerehabilitation after total knee replacement: preliminary results from Tuscany. Proceedings of the 3rd conference of IWBBIO: International Work – Conference on Bioinformatics and Biomedical Engineering; April 15-17, 2015; Granada, Spain. Switzerland: Springer International Publishing; 2015.

[9] García-Holgado A, Marcos-Pablos S, García-Peñalvo FJ. A model to define an eHealth technological ecosystem for caregivers. Proceedings of the 7th World Conference on Information Systems and Technologies; April 16-19, 2019; Galicia, Spain. Cham: Springer; 2019. [doi: 10.1007/978-3-030-16187-3_41]

[10] Grustam AS, Vrijhoef H, Cordella A, Koymans R, Severens JL. Care coordination in a business-to-business and a business-to-consumer model for telemonitoring patients with chronic diseases. International Journal of Care Coordination. 2017;20(4):135–147. [doi: 10.1177/2053434517747908]

[11] Grustam AS, Vrijhoef HJM, Koymans R, Poulikidis V, Severens JL. Extending the Business-to-Business (B2B) model towards a Business-to-Consumer (B2C) model for telemonitoring patients with Chronic Heart Failure (CHF). Journal of Business Models. 2018:6(3):106-129. [doi: 10.5278/ojs.jbm.v6i3.1596]

[12] Hwang J, Christensen CM. Disruptive innovation in health care delivery: a framework for business-model innovation. Health Affairs. 2008;27(5):1329–1335. [doi: 10.1377/hlthaff.27.5.1329]

[13] Kho J, Gillespie N, Horsham C, Snoswell C, Vagenas D, Soyer HP, et al. Skin doctor consultations using mobile teledermoscopy: exploring virtual care business models. Telemedicine and E-Health. 2020;26(11):1406–1413. [doi: 10.1089/tmj.2019.0228]

[14] Kijl B, Nieuwenhuis LJ, Huis in ’t Veld RM, Hermens HJ, Vollenbroek-Hutten MM. Deployment of e-health services – a business model engineering strategy. Journal of Telemedicine and Telecare. 2010;16(6):344–353. [doi: 10.1258/jtt.2010.006009]

[15] Kimble C. Business models for e‐health: evidence from ten case studies. Global Business and Organizational Excellence. 2015;34(4):18–30. [doi: 10.1002/joe.21611]

[16] Korsgaard F, Hasenkam JM, Vesterby M. Successful implementation of telemedicine depends on personal relations between company representatives and healthcare providers: a qualitative study of business models for Danish home telemonitoring. Health Services Management Research. 2021;34(4):223–233. [doi: 10.1177/0951484820988628]

[17] Kriegel J, Reckwitz L, Auinger K, Tuttle-Weidinger L, Schmitt-Rüth S, Kränzl-Nagl R. New service excellence model for eHealth and AAL solutions – a framework for continuous new service development. Health Informatics Meets eHealth. 2017. [doi: 10.3233/978-1-61499-759-7-275]

[18] Lee YL, Chang P. Modeling a mobile health management business model for chronic kidney disease. Nursing Informatics 2016. 2016;225:1047-1048. [doi: 10.3233/978-1-61499-658-3-1047]

[19] León MC, Nieto-Hipólito JI, Garibaldi-Beltrán J, Amaya-Parra G, Luque-Morales P, Magaña-Espinoza P, et al. Designing a model of a digital ecosystem for healthcare and wellness using the Business Model Canvas. Journal of Medical Systems. 2016;40(6):144. [doi: 10.1007/s10916-016-0488-3]

[20] Lin SH, Liu JH, Wei J, Yin WH, Chen HH, Chiu WT. A business model analysis of telecardiology service. Telemedicine and E-Health. 2010;16(10):1067–1073. [doi: 10.1089/tmj.2010.0059]

[21] Lin TC, Chang HJ, Huang CC. An analysis of telemedicine in Taiwan: a business model perspective. International Journal of Gerontology. 2011;5:189-192. [doi: 10.1016/j.ijge.2011.09.039]

[22] Martin A, Canu C, Breda G, Jean C. E-health deployment business model tele-expertise case study. Proceedings of the International Conferences e-Health 2018; ICT, Society, and Human Beings 2018; and Web Based Communities and Social Media 2018; July 17-19, 2018; Madrid, Spain.

[23] Mueller C. mHealth business model framework for the maternal and baby

segment: a design science research approach. Proceedings of the 32nd Bled eConference Humanizing technology for a sustainable society; Jun 16-19, 2019; Bled, Slovenia.

[24] Nikou S, Bouwman H. Mobile health and wellness applications: a business model ontology-based review. International Journal of E-Business Research. 2017;13(1),1–24. [doi: 10.4018/IJEBR.2017010101]

[25] Oderanti FO, Li F. Commercialization of eHealth innovations in the market of the UK healthcare sector: a framework for a sustainable business model. Psychology & Marketing. 2018;35(2):120–137. [doi: 10.1002/mar.21074]

[26] Oderanti FO, Li F, Cubric M, Shi X. Business models for sustainable commercialisation of digital healthcare (eHealth) innovations for an increasingly ageing population. Technological Forecasting and Social Change. 2021;171:120969. [doi: 10.1016/j.techfore.2021.120969]

[27] Pascarelli C, Colucci C, Mitrano G, Corallo A. Business models in digital health: bibliometric analysis and systematic literature review. 2023 IEEE Symposium on Computers and Communications (ISCC); Jul 09-12, 2023; Gammarth, Tunisia. [doi: 10.1109/ISCC58397.2023.10218237]

[28] Pereira SGM, Medina FADS, Gonçalves RF, Da Silva MT. System Thinking and Business Model Canvas for Collaborative Business Models Design. IFIP WG 5.7 International Conference, APMS 2016; Sept 3-7, 2016; Iguassu Falls, Brazil. Cham: Springer; 2016. [doi: 10.1007/978-3-319-51133-7_55]

[29] Peters C, Blohm I, Leimeister JM. Anatomy of successful business models for complex services: insights from the telemedicine field. Journal of Management Information Systems. 2015;32(3):75–104. [doi: 10.1080/07421222.2015.1095034]

[30] Pistorio A, Locatelli P, Cirilli F, Gestaldi L, Solvi S. A business model for digital healthcare environments: an organic approach and a use case for handling cognitive impairment. Proceedings of the 10th International Joint Conference on Biomedical Engineering Systems and Technologies (BIOSTEC 2017); Feb 21-23, 2017; Porto, Portugal. Setúbal: SCITEPRESS – Science and Technology Publications, Lda; 2017. [doi: 10.5220/0006168803400347]
[31] Radić M, Vienken C, Nikschat L, Dietrich T, Koenig H, Laderick L, et al. AI-based business models in healthcare: an empirical study of clinical decision support systems. Proceedings of the 7th International Conference on Digital Economy, ICDEc 2022; May 9–11, 2022; Bucharest, Romania. [doi: 10.1007/978-3-031-17037-9_5]

[32] Schuhmacher A, Haefner N, Honsberg K, Goldhahn J, Gassmann O. The dominant logic of Big Tech in healthcare and pharma. Drug Discovery Today. 2023;28(2):103457. [doi: 10.1016/j.drudis.2022.103457]

[33] Sprenger M. Supporting the viability of e-Health services with pattern-based business model design. Proceedings of the 6th International Conference on well-being in the information society, WIS 2016; Sept 16-18, 2016; Tampere, Finland. Cham: Springer; 2016. [doi: 10.1007/978-3-319-44672-1_14]

[34] Steinhauser S. Network-based business models, the institutional environment, and the diffusion of digital innovations: case studies of telemedicine networks in Germany. Schmalenbach Business Review. 2019;71(3):343–383. [doi: 10.1007/s41464-019-00076-9]

[35] Sterling R, LeRouge C. On-demand telemedicine as a disruptive health technology: qualitative study exploring emerging business models and strategies among early adopter organizations in the United States. Journal of Medical Internet Research. 2019;21(11):e14304. [doi: 10.2196/14304]

[36] Tageo V, Dantas C, Chronaki C, Lowe C, Berler A, Porcu F. Business Model Canvas insights for the adoption of international patient summary standards in the mhealth industry. Journal of Business Models. 2020;8(3):91-106. [doi: 10.5278/jbm.v8i3.3428]

[37] Thamjamrassri P, Song Y, Tak J, Kang H, Kong HJ, Hong J. Customer discovery as the first essential step for successful health information technology system development. Healthcare Informatics Research. 2018;24(1):79. [doi: 10.4258/hir.2018.24.1.79]

[38] Van Limburg M, Wentzel J, Sanderman R, Van Gemert-Pijnen L. Business modeling to implement an eHealth portal for infection control: a reflection on co-creation with stakeholders. JMIR Research Protocols. 2015;4(3):e104. [doi: 10.2196/resprot.4519]

[39] Van Meeuwen DP, Van Walt Meijer Q J, Simonse LW. Care models of eHealth services: a case study on the design of a business model for an online precare service. JMIR Research Protocols. 2015;4(1):e32. [doi: 10.2196/resprot.3501]

[40] Velayati F, Ayatollahi H, Hemmat M, Dehghan R. Key components and critical factors for developing a telehealth business framework: a qualitative study. BMC Medical Informatics and Decision Making. 2021;21(1):339. [doi: 10.1186/s12911-021-01707-3]

[41] Velayati F, Ayatollahi H, Hemmat M, Dehghan R. The 4P telehealth business framework for Iran. BMC Medical Informatics and Decision Making. 2022;22(1):266. [doi: 10.1186/s12911-022-02011-4]

[42] Velayati F, Ayatollahi H, Hemmat M, Dehghan R. Telehealth business models and their components: systematic review. J Med Internet Res. 2022;24(3):e33128. [doi: 10.2196/33128]

[43] Huis in 't Veld R, Fielt E, Vollenbroek-Hutten M. Moving telemonitoring and -treatment from promise to practice: a business model approach for a chronic lower back pain application. International Journal of Healthcare Technology and Management. 2011;12(3/4):333-349. [doi: 10.1504/IJHTM.2011.040483]

[44] Visser JJ, Bloo JK, Grobbe FA, Vollenbroek-Hutten MM. Video teleconsultation service: who is needed to do what, to get it implemented in daily care? Telemed J E Health. 2010;16(4):439-45. [doi: 10.1089/tmj.2009.0101]

[45] Viswanadham N. Ecosystem model for healthcare platform. Sādhanā. 2021;46(4):188. [doi: 10.1007/s12046-021-01708-y]
