# Supplementary material for: A Business Model Framework for Software as a Medical Device Startups in the European Union: Mixed Methods Study
Source: J Med Internet Res. 2025 May 23;27:e67328. doi: 10.2196/67328 (PMC12144475; doi:10.2196/67328)
Supplement: Multimedia Appendix 3 [file jmir_v27i1e67328_app3.docx]

Multimedia Appendix 3. Sample of experts for interviews.

| **ID** | **Organization** | **Role** | **Duration** |
| --- | --- | --- | --- |
| IN1 | SaMD Startup | Co-Founder and CEO | 31 min |
| IN2 | SaMD Startup | Founder and CEO | 36 min |
| IN3 | SaMD Startup | Co-Founder and CEO | 26 min |
| IN4 | SaMD Startup | Founder and CEO | 50 min |
| IN5 | SaMD Startup | CEO and Managing Director | 27 min |
| IN6 | SaMD Startup | Co-Founder and CMO | 23 min |
| IN7 | SaMD Startup | CTO | 22 min |
| IN8 | Accelerator | Startup Coach and Founder | 66 min |
| IN9 | Startup^a^ | Co-Founder and CEO, VP Product & Marketing^b^ | 55 min |
| IN10 | SaMD Startup | CEO and Co-Founder | 40 min |
| IN11 | SaMD Startup | Senior Business Development Manager | 70 min |
| IN12 | Incubator, Accelerator | Head of the incubator, accelerator | 33 min |
| IN13 | Pharmaceutical Company | Start-up and Digital Innovation Scout, Founder | 28 min |
| **Total** |  |  | **507 min** |

^a:^ Considering changing product focus slightly, therefore qualifying as a SaMD

^b^: Interview was conducted with two employees at the same time
