# Supplementary material for: A Business Model Framework for Software as a Medical Device Startups in the European Union: Mixed Methods Study
Source: J Med Internet Res. 2025 May 23;27:e67328. doi: 10.2196/67328 (PMC12144475; doi:10.2196/67328)
Supplement: Multimedia Appendix 6 [file jmir_v27i1e67328_app6.docx]

Multimedia Appendix 6. Overview of inductive coding according to Gioia et al [1].

**1st Order Concept 2nd Order Themes Aggregated Dimensions**

Value proposition for each customer segment

- Value proposition from all angles
- Value proposition is difficult to define for

health insurance company

- Offering product with certain safety standards
- Offering quality to customer through certification

Value offering

- Health insurance company as payer in case of reimbursement and individual patient as potential user
- Doctors as prescribers

User, payer, prescriber

- Which customer to address?
- Who is the payer and who is the end user of the product?

Customer identification

- Functioning of the product
- Lifestyle product vs. medical device

Device characteristics

- Product changes or updates of certified product require recertification
- Digital products are evolving and require updates

Device adaptions

- Risk classification depends on intended purpose
- Intended purpose needs to be set up to provide value

Intended purpose

- MDR requires a lot of experience
- Lack of transparency about what is expected
- MDR entails a sea of documents
- MDR needs to be considered from the

beginning on

- MDR as the crucial point

Challenges

MDR from the very beginning

- Building and training a sales team
- Marketing

Activities not directly resulting from MDR

Activities directly resulting from MDR

- QMS as a huge topic (full time job)
- Clinical investigation as a difficult and lengthy path
- Regulatory expertise required (Regulatory

Affairs Person)

- Quality manager required
- Person with experience in bringing medical devices to the market

Human Resources

- Clinical data
- Health data is valuable and should be protected

Data

- Who will pay for the product (e.g., self-payment, health insurance company, hospitals)?
- Health insurance company as a common

revenue source in Germany (reimbursement)

Revenue sources

- Revenue model depends on country and product (offered revenue pathways such as DiGA)
- Revenue model depends on product and the market for the product (for some products there is a self-payment willingness and for others not)

Type of revenue model

**1st Order Concept 2nd Order Themes Aggregated Dimensions**

- Consulting companies which support in MDR-related matters
- Costs for notified bodies
- Clinical investigation is costly and takes long time
- Ongoing IT costs
- Marketing
- Sales people

Costs directly arising from MDR

Costs not directly arising from MDR

- Understanding the healthcare market itself and how it works
- Possibly entering the market earlier with an adapted version of the product
- Entering the market earlier in a less regulated country

Understanding the healthcare market

- Unique Selling Propositions should be properly worked out early on
- Potential differentiation to competitors via well-functioning customer support service

Competitive advantage

- Communication channels used by the health insurance company with its insured individuals foster trust
- Sales teams that distribute device to doctors

Channel selection

Importance of distribution channels

Importance of partnerships

Partner selection

- Important to have alliances with other people, otherwise you will not survive in the digital health sector
- Getting in touch with other people and working together is crucial
- Not everything should be handed over to external parties
- Pharmaceutical companies offer access to patients
- Consulting companies can support in

regulatory matters

- Cooperation with health insurance company brings credibility
- Strategic distribution planning in respective target countries
- Setup of a product. And distribution channel management matrix for international business
- Many investors do not accept the delay of the first revenues due to time consuming CE

making process

- Difficult to attract investors without generating revenues
- Startups are fighting financially survive until market entry
- Follow-up funding early on

Investors

Financial resources

**1st Order Concept 2nd Order Themes Aggregated Dimensions**

- Lock-in effect can make a business model in digital health powerful
- How to maintain customers?
- Customer relationships are essential
- Engage with customers early to gather

feedback

Lock-in effect

Importance of customer relationships

- Identification of stakeholders and relations between stakeholders
- Complex stakeholder constellation
- Who are the biggest players?
- Interest of stakeholders
- Understand the language of the actors
- How does a health insurance company work?

Stakeholder constellation

Characteristics of stakeholders

**References**

[1] Gioia DA, Corley KG, Hamilton AL. Seeking qualitative rigor in inductive research: notes on the Gioia methodology. Organ Res Methods. Jul 24, 2012;16(1):15-31. [doi: 10.1177/1094428112452151]
