# Supplementary figures and images for: A Business Model Framework for Software as a Medical Device Startups in the European Union: Mixed Methods Study
Source: J Med Internet Res. 2025 May 23;27:e67328. doi: 10.2196/67328 (PMC12144475; doi:10.2196/67328)

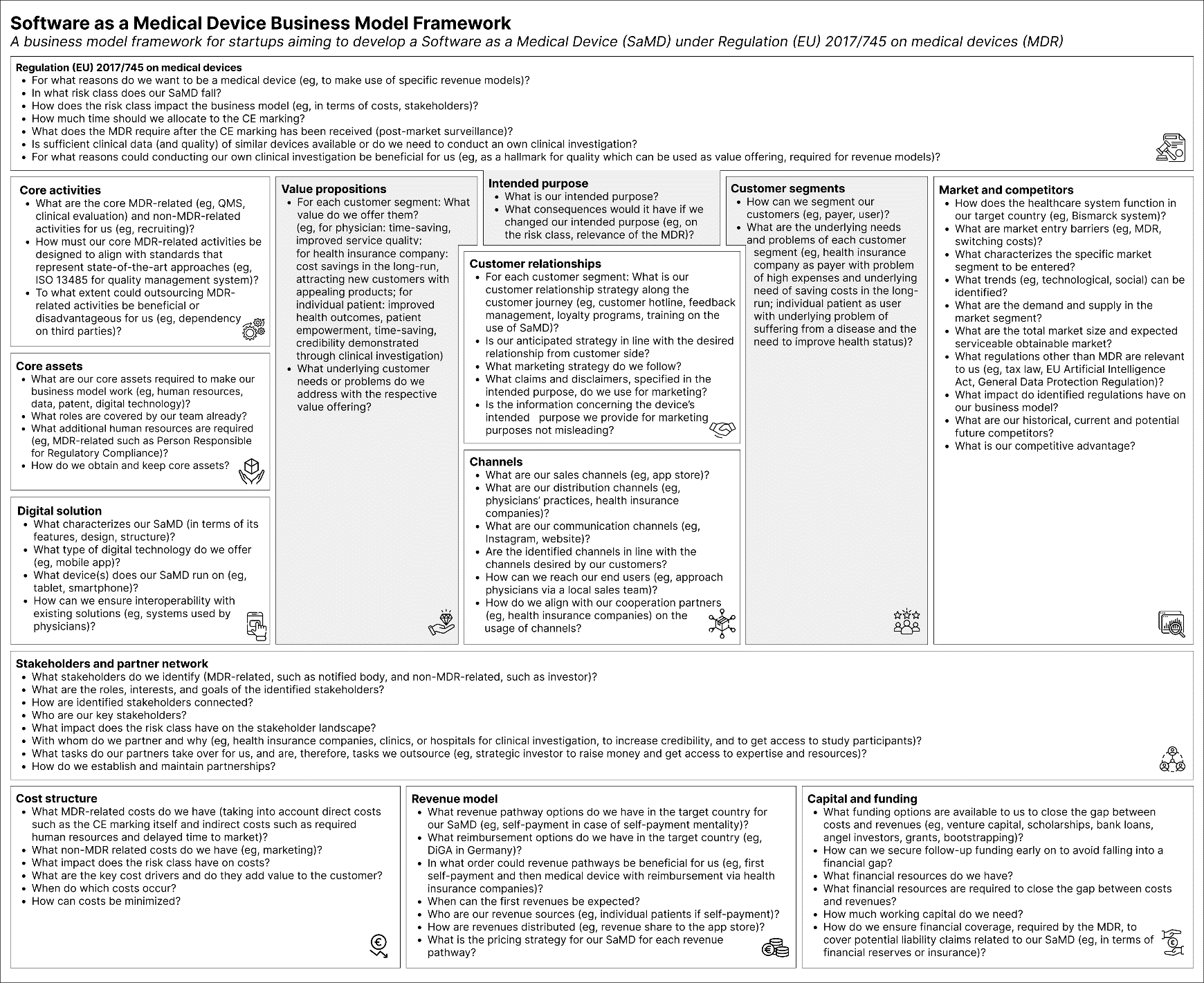
Multimedia Appendix 7. Software as a Medical Device Business Model Framework.

Supplement: Multimedia Appendix 7 [file jmir_v27i1e67328_app7.docx]
