# Supplementary material for: A Business Model Framework for Software as a Medical Device Startups in the European Union: Mixed Methods Study
Source: J Med Internet Res. 2025 May 23;27:e67328. doi: 10.2196/67328 (PMC12144475; doi:10.2196/67328)

Multimedia Appendix 8. Exemplary filled-out Software as a Medical Device Business Model Framework.


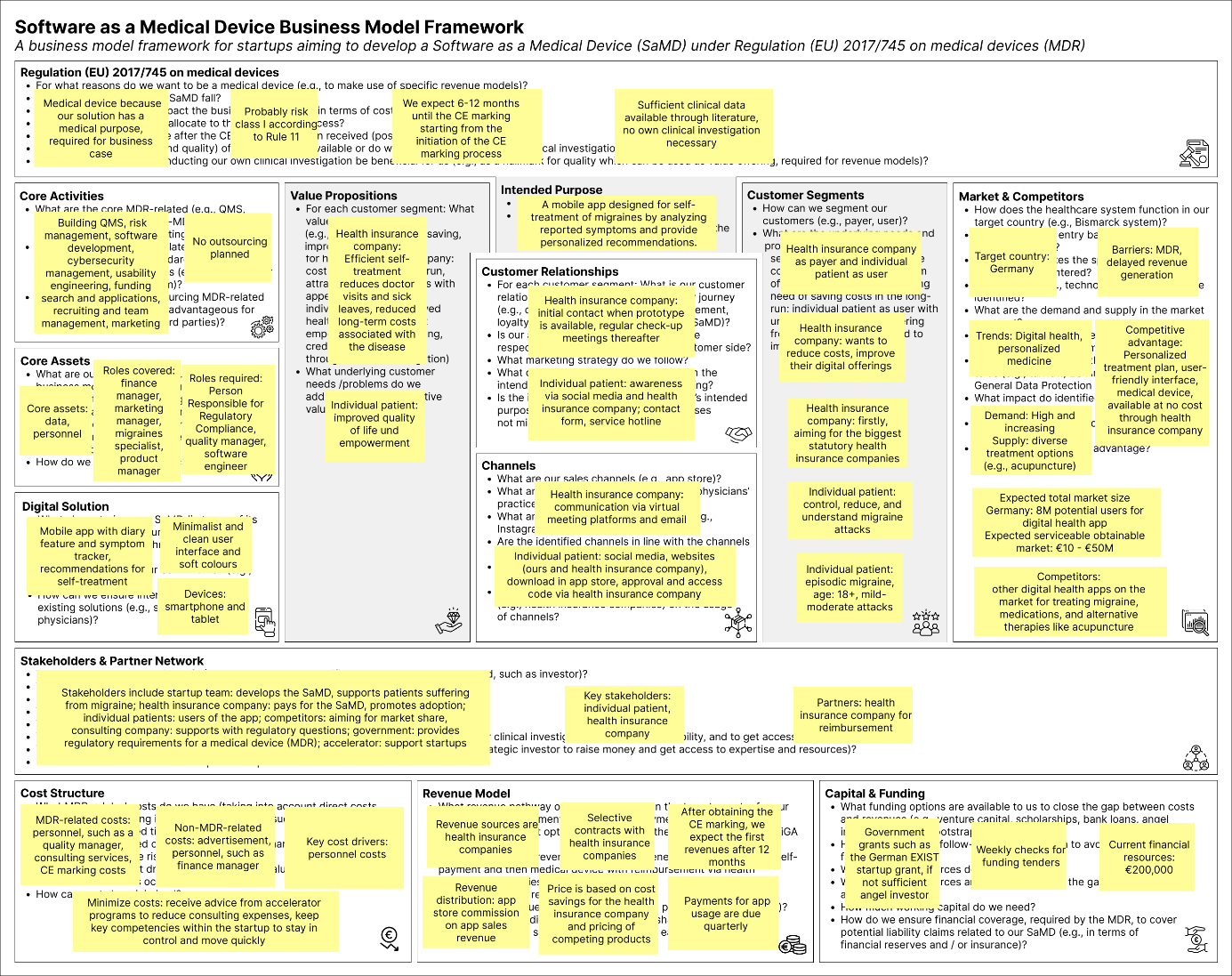

Supplement: Multimedia Appendix 8 [file jmir_v27i1e67328_app8.docx]
